# Evolving Treatment Landscape for Advanced Esophageal and Gastroesophageal Junction Adenocarcinoma

**DOI:** 10.1007/s11912-024-01607-5

**Published:** 2024-10-23

**Authors:** Margaret C. Wheless, Margaret Comer, Michael K. Gibson

**Affiliations:** 1 Department of Medicine, Division of Hematology Oncology, Vanderbilt University Medical Center, Nashville, TN USA; 2 Vanderbilt University Medical School, Nashville, TN USA; 3 Vanderbilt Ingram Cancer Center, Vanderbilt University Medical Center, 2220 Pierce Avenue, 777 Preston Research Building, Nashville, TN 37232 USA

**Keywords:** Esophageal Adenocarcinoma, Gastroesophageal Junction Adenocarcinoma, Claudin18.2, FGFR, HER-2, PD-L1 Therapy

## Abstract

**Purpose of Review:**

This review highlights advances and recent changes in the treatment paradigm for advanced esophageal adenocarcinoma (EAC) and gastroesophageal junction adenocarcinoma (GEJAC).

**Recent Findings:**

Chemotherapy remains the backbone of treatment for advanced EAC/GEJAC. New targets/agents include immunotherapy, HER-2, claudin18.2, and FGFR2b, with various mechanisms (CAR-T, bispecific mAB, ADCs) altering the treatment landscape against these targets.

**Summary:**

The approaches to these targets may act together, in sequence, and even synergistically to improve outcomes. Herein, we review the state of the field, including highlighting ongoing clinical trials and additional emerging agents and approaches.

## Introduction

The treatment paradigm for EAC and GEJAC has steadily changed within the last several years with the incorporation of immunotherapy and targeted therapy. Though there have been significant advances, the overall prognosis for patients with advanced disease remains poor. As more therapies are developed for targetable cancer antigens, mutational profiling will become increasingly important as well as sequencing of targeted therapy. Herein, we hope to highlight the evolution of treatment for advanced EAC and GEJAC, expound on novel treatments, and emphasize the importance of clinical trial enrollment and mutational profiling to ultimately improve outcomes in this patient population.

## Epidemiology and Risk Factors

Esophageal cancer (EC) represents a significant source of morbidity and mortality worldwide, accounting for approximately 604,100 new cases and 544,100 deaths in 2020 [1]. Globally, it ranks seventh in incidence and sixth in mortality compared to other types of cancer. The age-standardized incidence rates are 2.5-fold higher in men than women, although that varies considerably by population [2]. Over the past four decades, there has been a considerable shift in the regional incidence of the different subtypes of esophageal cancer. Previously, squamous cell carcinoma (SCC) comprised the vast majority of EC, but EAC now predominates in several high-income countries, including the United States, the United Kingdom, Australia, and France. SCC rates are highest in Eastern Asia, Southern Africa, Eastern Africa, and parts of Northern Europe [3]. In contrast, EAC accounts for 64% of EC in the United States, compared to 31% SCC and 5% other types [4].

In the U.S., the incidence of EAC increased from 3.6 cases per million to 25.6 cases per million between 1973–2006, although incidence rates appear to have stabilized or decreased between 1997–2014 with some variation between sexes, ethnic groups, and age groups [5, 6]. The incidence of GEJAC increased 2.5-fold between the 1970s-1990s and later stabilized, although the exact incidence is difficult to ascertain due to inconsistencies in classification systems in defining EAC versus GEJAC [7]. These shifting epidemiologic trends likely reflect the rise and fall of certain risk factors in different populations. Regardless of subtype, EC/GEJAC has a high mortality rate, with a five-year survival rate of less than 20%. Patients often present with advanced disease, with up to half of patients demonstrating metastases at the time of diagnosis. Most patients initially diagnosed with local regional disease will eventually develop metastases after treatment for curative intent [8].

Established risk factors for SCC include smoking, alcohol consumption, frequent consumption of hot food and beverages, achalasia, and diets heavy in nitrosamine-containing compounds [2]. While smoking is a common risk factor for both subtypes, the decline in SCC in high-income Western countries is largely attributed to the decline in cigarette smoking [3]. Unique risk factors for EAC and GEJAC include gastroesophageal reflux disease and obesity, although the association is weaker for GEJAC [7]. The metaplastic precursor lesion to EAC, Barrett’s esophagus, results from chronic reflux of stomach acid and bile salts with resultant inflammation. Rising rates of EAC coincide with increasing obesity in Western countries. Increased central adiposity can mechanically contribute to reflux through higher intragastric pressure and lower esophageal sphincter (LES) dysfunction [9]. Obesity may also contribute to EAC through reflux-independent, hormonally-mediated mechanisms. Increased adipose tissue leads to higher leptin secretion and decreased adiponectin secretion, which have been shown to synergistically promote malignant behavior in experimental esophageal cell lines [10]. A Western diet low in fruits and vegetables and high in fat and processed meat may also contribute to EAC development [11]. Medications implicated in transient relaxation of the LES, such as anticholinergics, may increase risk, although there is mixed evidence [12]. An additional potential risk factor which may be contributing to the rise of EAC is the falling rate of H. Pylori infection and associated atrophic gastritis in Western countries. There is a hypothesized protective effect due to less acidic gastric juices in individuals infected with H. Pylori, which may induce less damage to the esophageal tissue. Epidemiological studies in multiple countries demonstrate an inverse relationship between the incidence of gastric adenocarcinoma, of which H. Pylori infection is a known risk factor, and EC. Eradication of H. Pylori is also associated with weight gain, which may potentiate the effects of obesity on EAC development. Although not the singular cause behind the rise of EAC, the loss of protective effect against damaging reflux disease, in the context of increasing rates of central obesity, may be a potent driver behind these changing epidemiologic trends [13, 14].

## Chemotherapy and Immunotherapy

### First Line Chemotherapy and Immunotherapy

The backbone of therapy for metastatic EC and GEJAC remains cytotoxic chemotherapy (summarized in Table 1). Initial doublet chemotherapy backbones consisted of 5-fluorouracil (5FU) and cisplatin (CF) which were established for esophageal SCC and extrapolated to EAC in 1997 [15]. As more chemotherapy options were developed, other agents were added to improve efficacy. Triplet combinations including taxanes (Docetaxel/CF) or anthracyclines (Epirubicin/CF) were more toxic than doublets but with similar efficacy [16, 17]. In the phase III REAL-2 study, oxaliplatin was compared to cisplatin in the epirubicin triplet and found to be noninferior to cisplatin with less grade 3/4 neutropenia, renal disease, and thromboembolism [17]. Based on these findings, 5FU in combination with oxaliplatin was initially evaluated in a phase III study conducted in Germany which enrolled patients with treatment-naïve GC or GEJ cancer (N = 220; GEJ, n = 44) to FLO (5FU, leucovorin, oxaliplatin) or FLP (5FU, leucovorin, cisplatin) and found improved tolerability with FLO with significantly less grade 3/4 anemia (p < 0.001), leukopenia (p = 0.022), nausea (p = 0.003), and vomiting (p = 0.002). The duration of treatment was significantly longer with FLO compared to FLP (5 vs 3.1 mo; p = 0.003), and though not powered for OS comparison, the mOS was 10.5 months (FLO) compared to 8.8 months (FLP) [18]. This established oxaliplatin as the preferred platinum moving forward.

**Table 1:** Clinical trials highlighting first and second-line chemoimmunotherapy

NCT/Reference	Trial	Study Agent	Study Population	Primary Outcome	mPFS or mTTF (months)	HR (95% CI) p-value	mOS (months)	HR (95% CI) p-value	ORR (%)
*First Line Chemotherapy and Immunotherapy*									
ISRCTN51678883 [17]	REAL-2	ECF or ECX vs EOF or EOX	1st line: GC, GEJ, EC (SCC, EAC)	OS	mPFS: ECF 6.2, ECX 6.7, EOF 6.5, EOX 7.0		ECF 9.9, ECX 9.9, EOF 9.3, EOX 11.2	capecitabine vs 5FU: 0.86 (0.80, 0.99) oxaliplatin vs cisplatin: 0.92 (0.80, 1.10)	ECF 40.7%, ECX 46.4%, EOF 42.4%, EOX 47.9%
[18]		FLO vs FLP	1st line, GC, GEJAC	PFS	mPFS: 5.8 vs 3.9	p = 0.077	10.7 vs 8.8	p = 0.506	
[19]		FOLFOX	2nd or later line EC (EAC, ESCC)	ORR	mPFS: 4.6	2.2–6.8	7.1	5.9–10.9	40%
[20]		Paclitaxel + cisplatin	1st line, ESCC and EAC	ORR	Allcomers: 5.7 EAC: 5.7	1.0 – 18.6	Allcomers: 10.8 EAC: 10.0	Allcomers: 1.5 –25 + EAC 1.6 – 25 +	Allcomers: 48% EAC: 34%
[21]		Paclitaxel	1st line, ESCC and EAC	ORR			13.2	2 – 17.5 +	Allcomers: 32% EAC: 34%
NCT02872116 [22]	CheckMate 649	nivo + chemo (FOLFOX or XELOX) vs chemo	1st line HER-2 negative GC, GEJ, or EAC	OS and PFS	mPFS: CPS ≥ 5: 7.7 vs 6.05	CPS ≥ 5: 0.68 (98% CI 0.56, 0.81)	CPS ≥ 5: 14.4 vs 11.1	CPS ≥ 5: 0.71 (0.59, 0.86) p < 0.0001	CPS ≥ 5: 60% vs 45%
NCT03189719 [23]	KEYNOTE-859	Pembro + chemo (FP) vs placebo + chemo	1st line ESCC, EAC, GEJAC	OS	mPFS: 6.9 vs 5.6 CPS ≥ 10: 8.1 vs 5.6	0.75 (0.67, 0.85) p < 0.0001 CPS ≥ 10: 0.62 (0.51, 0.76) p < 0.0001)	Allcomers: 12.9 vs 11.5 CPS ≥ 10: 15.7 vs 11.8	0.78 (0.70, 0.87) p < 0.0001 CPS ≥ 10: 0.65 (0.53, 0.79) p < 0.0001	45.0% vs 29.3%
NCT03189719 [24]	KEYNOTE-590	pembro + chemo (FP) vs chemo	1st line EC (ESCC, EAC) or GEJAC	OS and PFS	mPFS: 6.3 vs 5.8 (allcomers) EAC: 6.3 vs 5.7	0.65 (0.55, 0.76) p < 0.0001 EAC: 0.63 (0.46, 0.87)	12.4 vs 9.8 (allcomers) EAC: 11.6 vs 9.9	0.73 (0.62, 0.86) p < 0.0001 EAC: 0.74 (0.54, 1.02)	45.0% vs 29.3%
NCT00374036 [25]		FOLFIRI vs ECX	1st line, GC, GEJ	TTF	mTTF: 5.1 vs 4.2	0.77 (CI 0.63, 0.93) p = .008	9.7 vs 9.5	1.01 (CI 0.82, 1.24) p = 0.95	37.8% vs 39.2%
[26]		Carboplatin + paclitaxel	1st line, ESCC or EAC	ORR			9.0	(90% CI 7, 13.8)	43%
NCT02628067 [27]	KEYNOTE-158	Pembro	dMMR/MSI-H non-CRC (including GC)	ORR	GC mPFS: 11	2.1-NE	GC: 19.8	14.5–25.8	GC: 45.8%
*Second and Later Line Chemotherapy and Immunotherapy*									
[28]		Irinotecan + 5-FU + leucovorin	2nd line, ESCC, EAC, GEJAC	ORR	mPFS: 3.7	2.3–5.1	6.4	3.2–6.9	29%
[29]		Irinotecan vs Irinotecan + mFOLFIRI	2nd line, GC, GEJAC	ORR	mPFS: 2.2 vs 3.0	1.20 (0.72, 2.02) p = 0.481	5.8 vs 6.7	1.21 (0.69, 2.11) p = 0.514	17.2% vs 20.0% p = 0.525
NCT00144378 [30]		Irinotecan vs BSC	2nd line, GC, GEJAC	OS	Irinotecan group: mPFS: 2.5	1.6–3.9	4.0 vs 2.4	0.48 (0.25, 0.92) p = 0.012	No objective responses
NCT01170663 [31]	RAINBOW	Ramucirumab + paclitaxel vs placebo + paclitaxel	2nd line, GC, GEJAC	OS	mPFS: 4.4 vs 2.9	0.64 (0.54, 0.75) p < 0.0001	9.6 vs 7.4	0.81 (95% CI 0.68, 0.96) p = 0.017	28% vs 16% p = 0.0001
NCT00917384 [32]	REGARD	Ramucirumab vs placebo	2nd line, GC, GEJAC	OS	mPFS: 2.1 vs 1.3	0.48 (0.376, 0.620) p < 0.0001	5.2 vs 3.8	0.78 (95% CI 0.603–0.998) p = 0.047	
NCT02559687 [33]	KEYNOTE-181	Pembro vs paclitaxel, docetaxel, or irinotecan	2nd line, ESCC, EAC, or GEJAC	OS	mPFS: 2.1 vs 3.4	1.11 (0.94, 1.31)	Allcomers: 7.1 vs 7.1 CPS ≥ 10: 9.3 vs 6.7	Allcomers: 0.89 (0.75, 1.05) p = 0.056 CPS ≥ 10: 0.69 (0.52, 0.93) p = 0.0074	13.1% vs 6.7%
NCT02715284 [34]	GARNET	Dostarlimab	advanced dMMR-MSI-H solid tumors, GC/GEJAC cohort	ORR	Allcomers: mPFS: 6.9 GC/GEJAC mPFS: 5.5	Allcomers: 4.2–13.6 GC/GEJAC: 2.8-NE	Allcomers: NR GC/GEJAC: 20.1	Allcomers: n/a GC/GEJAC: 6.7-NE	Allcomers: 44.0% GC/GEJAC: 45.5%
NCT02500043 [35]	TAGS	Trifluridine/ tipiracil vs placebo	3rd line GC, GEJAC	OS	mPFS: 2.0 vs 1.8	0.57 (0.47, 0.70) p < 0.0001	5.7 vs 3.6	0.69 (0.56, 0.85) p = 0.0003	4% vs 2%

Trials highlighting first and second-line chemoimmunotherapy options. In select trials, subgroup analysis for cohort of interest is reported. Abbreviations: *BSC* best supportive care, *HR* hazard ratio, *mOS* median overall survival, *mPFS* median progression free survival, *mTTF* median time to treatment failure, *ORR* objective response rate

For patients unable to tolerate platinum agents due to renal insufficiency or neuropathy, FOLFIRI (5FU plus irinotecan) is an option. In a French phase III study, 416 patients (GC, n = 276; GEJ, n = 136) with advanced disease were randomized to either epirubicin, cisplatin, and capecitabine (ECX) or FOLFIRI with crossover at progression. Though the FOLFIRI cohort had significantly longer time to treatment failure (5.1 vs 4.2mo; HR 0.77 [95% CI 0.63 – 0.93]; p = 0.008), there was no significant difference between ECX and FOLFIRI regarding mPFS (5.3 vs 5.8mo; HR 0.99), mOS (9.5 vs 9.7mo; HR 1.01), or ORR (39.2% vs 37.8%)—likely as a result of crossover. There were significantly less grade 3/4 AEs compared to ECX (p < 0.001) and thus FOLFIRI may be considered in the first line setting for patients who are platinum ineligible [25].

Paclitaxel first proved efficacious in a phase II study of 50 treatment-naïve patients (EAC, n = 32; ESCC, n = 18) with advanced EC in 1994. The mOS of the entire cohort was 13.2 months (range 2 to > 17.5 months), and the 32 patients with EAC had an ORR of 34% (95% CI 18 – 50%) [20]. It’s efficacy and tolerability led to a phase II trial combining cisplatin with paclitaxel in the first-line setting in 38 patients with EC/GEJ cancer. The ORR was 46% in patients with adenocarcinoma (n = 33) with mOS of 6.9 months. Unfortunately, 11% of patients experienced death due to treatment-related toxicity, and 50% were hospitalized due to AEs [21]. Because this regimen proved highly toxic, other tolerable and efficacious combination therapies were needed. Paclitaxel, combined with carboplatin, was evaluated in a phase II trial. Patients with both SCC (n = 13) and adenocarcinoma (n = 22) histology of esophageal (n = 25) and GEJ (n = 10) origin were included and had an ORR of 43% (90% CI 0.31 – 0.61) with mPFS of 9 months (90% CI 7.0 – 13.8). There was no significant difference in response rates between patients with adenocarcinoma or squamous histology and paclitaxel + carboplatin was generally well tolerated with grade 3/4 AEs of neutropenia (n = 17; 2 with neutropenic fever) and anemia (n = 6) [26].

The standard of care changed with the emergence of targeted agents including immune checkpoint inhibitors (ICI) as a new paradigm for cancer immunotherapy. Two such monoclonal antibodies that block the PD1/PD-L1 interaction by binding the PD-1 receptor, pembrolizumab (pembro) and nivolumab (nivo), increase survival when added to chemotherapy. The phase III CheckMate 649 study compared in the first-line recurrent/metastatic (R/M) setting nivo plus chemotherapy (FOLFOX or XEL(xeloda)OX) to chemotherapy alone in 1581 patients with metastatic GC (n = 1110), GEJAC (n = 260), and EAC (n = 211). The dual primary endpoints were OS and PFS in patients with CPS ≥ 5. All patients were HER-2 negative and were stratified by Combined Prognostic Score (CPS evaluates the number of PD⁠-⁠L1–staining cells (tumor cells, lymphocytes, macrophages) relative to all viable tumor cells) of which 60% (chemo plus nivo) and 61% (chemo) of patients had CPS ≥ 5. In patients with CPS ≥ 5, the addition of nivo was significantly beneficial with improved mOS (14.4 vs 11.1 mo; HR 0.71 [95% CI 0.59 – 0.86]; p < 0.0001) and mPFS (7.7 vs 6.05 mo). In patients with CPS < 5 (HR 0.94 [95% CI 0.78 – 1.13]) and CPS < 1 (HR 0.92 [95% CI 0.70 – 1.23]), the mOS benefit with nivo was not statistically significant between the two cohorts. Patients with CPS ≥ 5 achieved the most benefit with the addition of ICI [22]. In a 3-year follow up in patients with CPS ≥ 5, the ORR was improved at 60% vs 45% with the addition of nivo with duration of response (DOR) of 9.6 months vs 7.0 months with chemo alone [36]. The addition of nivo to chemotherapy was generally well-tolerated with similar amounts of grade 3/4 neutropenia (15%, nivo + chemo; 12%, chemo) and anemia (6%, nivo + chemo; 3%, chemo) [22]. As such, the NCCN approved nivo in addition to chemotherapy in the first line as a category 1 recommendation for CPS ≥ 5 and category 2B for CPS < 5 [37].

The phase III KEYNOTE-590 study compared 749 patients (EAC, n = 111; GEJ, n = 91; SCC, n = 548) receiving pembrolizumab (pembro, anti-PD1 antibody) plus chemo (5FU plus cisplatin, FP) or chemo alone. Though most had ESCC, in a prespecified subgroup analysis, there was improved mPFS with the addition of pembro to chemo (6.3 vs 5.7 months; HR 0.63 [95% CI 0.46.- 0.87]) and a trend towards improved mOS (11.6 vs 9.9 months; HR 0.74 [95% CI 0.54 – 1.02]) in the adenocarcinoma cohort. All patients showed improved outcomes with the addition of pembro, but the most benefit was seen in patients with CPS ≥ 10 (mOS HR 0.62 [95% CI 0.49 – 0.78]; mPFS HR 0.51 [95% CI 0.41 – 0.65]). Pembro was well-tolerated with similar rates of grade 3/4 neutropenia (14%, pembro + FP; 16%, FP) and anemia (12%, pembro + FP; 15%, FP) [24].

KEYNOTE-859 similarly compared pembro plus chemotherapy (FP or FOLFOX) versus chemo alone but in 1579 patients with metastatic gastric (n = 1243) and GEJ adenocarcinoma (n = 334). The mOS was significantly prolonged with the addition of pembro (12.9 vs 11.5 months; HR 0.78 [95% CI 0.7 – 0.87]; p < 0.0001) and even more striking in patients with CPS of ≥ 10 (15.7 vs 11.8 months; HR 0.65 [95% CI 0.53.- 0.79]; p < 0.0001). The mPFS was also improved with pembro in all comers (6.9 vs 5.6 months; HR 0.75 [95% CI 0.67 0. 0.85[; p < 0.0001) with the most benefit seen in those with CPS ≥ 10 (8.1 vs 5.6 months; HR 0.62 [95% CI 0.51 – 0.76]; p < 0.0001).Grade 3/4 potentially irAEs of colitis (2.4%) and skin reaction (2%) were more common in the pembrolizumab arm [23]. Given these findings, the addition of pembro to chemo in the first line in patients with CPS ≥ 10 is a category 1 recommendation in the NCCN guidelines but remains category 2B in patients with CPS < 10 [37].

Treatment with ICI is also an option for patients with mismatch repair deficient (dMMR) or microsatellite instability (MSI) high tumors. Though rare, with an estimated prevalence of just 3.8% in EC and 8.7% in GC, MSI or MMR testing is recommended for all patients at diagnosis [37, 38]. The phase II KEYNOTE-158 trial enrolled patients with dMMR/MSI-H non-colorectal cancer to receive pembrolizumab for up to 2 years in the second or later line. A large majority of patients enrolled had GC (n = 24, 10.3%) and though there were 27 different tumor types included, no patients had EAC. In the GC cohort, ORR was 45.8% (95% CI 25.6 – 67.2) and mPFS was 11 months (95% CI 2.1 – NE) [27]. In an updated analysis, the mOS for the study was 19.8 months (95% CI 14.5 – 25.8) [39]. Pembrolizumab was generally well-tolerated with 14.6% of patients experiencing grade 3 or more AEs, the most common being pneumonitis (3%) and skin reactions (3%). After this trial, pembrolizumab was given a tumor-agnostic approval for patients with dMMR/MSI-H solid tumors in the first-line given its efficacy in dMMR solid tumors, regardless of type [40, 41].

### Second and Later Line Chemotherapy and Immunotherapy

FOLFOX plus ICI is typically preferred in the first line, so FOLFIRI, VEGFR inhibition, or taxane-based therapy are reserved for the second line [25]. Several phase II have evaluated the efficacy of FOLFIRI after progression on platinum-based therapy showing tolerability with modest PFS (mPFS ranging from 3–3.7 months) and OS (mOS randing from 6.4 – 6.7 months) benefits and ORR of 20–29% [28, 29]. Irinotecan monotherapy in the second or later-line has also shown improved OS (4 vs. 2.4 months; HR 0.48; p – 0.012) when compared to best supportive care (BSC) in a phase III trial [30]. Though this study was done in patients with gastric cancer, treatment is extrapolated to EAC/GEJAC patients and remains a standard option within NCCN guidelines [37].

In the REGARD trial, ramucirumab (ram, anti-VEGFR-2 antibody) monotherapy with BSC was compared to BSC + placebo in the second or later-line setting in this phase III trial. In the 335 patients enrolled (GC, n = 265; GEJ, n = 90) 2:1 to ram + BSC or placebo + BSC, ram provided a modest mOS (5.2 vs 3.8mo) and mPFS benefit (2.1 vs 1.3mo). The mPFS (HR 0.39 [95% CI 0.23 – 0.65]) was significantly improved in the GEJ subgroup compared to GC with a trend towards improved mOS (HR 0.76 [95% CI 0.47 – 1.21]). Ramucirumab monotherapy was tolerated well with less grade 3/4 fatigue (6% vs 10%) and anemia (8% vs 6%) but with increased rates of HTN (8% vs 3%), and similar rates of bleeding (3%) [32]. For patients who may have comorbidities preventing combination chemotherapy or are limited by performance status but remain treatment-minded, ram monotherapy may be considered.

The combination of paclitaxel with ram was studied in the phase III RAINBOW trial which enrolled 665 patients (GC, n = 528; GEJ, n = 137) to either ram + paclitaxel or placebo + paclitaxel in the second or later-line. Though the majority of patients had GC, this data has been extrapolated to EAC given the significant improvement in mOS (9.6 vs 7.4mo; HR 0.81 [95% CI 0.68 – 0.96]; p = 0.017), mPFS (4.4 vs 2.9mo; HR 0.64 [95% CI 0.54 – 0.72]; p < 0.001), and ORR (28% vs 16%; p = 0.001) with combination ram plus paclitaxel. This is a category 1 recommendation for GEJ cancer and category 2B in EAC according to NCCN [31, 37]. In a preplanned subgroup analysis, patients with GEJ, compared to GC, had significantly improved mOS (HR 0.52 [95% CI 0.35 – 0.78]) and mPFS (HR 0.39 [95% CI 0.26 – 0.59]). More grade 3/4 fatigue (12% vs 5%), neuropathy (8% vs 5%), hypertension (14% vs 2%), and neutropenia (40% vs 7%) were reported in the combination group compared to paclitaxel monotherapy but with no new safety concerns [31].

Immunotherapy, if not given in the first-line setting, has also been evaluated as monotherapy in the second-line or later in the phase III KEYNOTE-181 trial which randomized 628 patients with EC (EAC, n = 227) to pembrolizumab or chemotherapy (investigator’s choice: docetaxel, paclitaxel, or irinotecan) regardless of CPS score (CPS < 10, n = 397; CPS ≥ 10, n = 222). The mOS was 7.1 months (HR 0.89 [95% CI 0.75 – 1.05]; p = 0.056) in both groups. In the CPS ≥ 10 cohort the pembrolizumab arm was superior (mOS 9.3 vs 6.7 months; HR 0.69 [95% CI 0.52 – 0.93]; p = 0.0074). Further subgroup analysis revealed that patients with ESCC and CPS ≥ 10 derived the most benefit from pembro (HR 0.64 [95% CI 0.46 – 0.90]) compared to EAC and CPS ≥ 10 (HR 0.93 [95% CI 0.52 – 1.65]) [33]. Pembrolizumab monotherapy may be considered for patients who did not receive ICI in the first line setting with CPS ≥ 10, though it’s efficacy may be less in those with EAC compared to ESCC.

Another ICI, dostarlimab (anti-PD-1 mAb), is approved in the second or later line for patients with dMMR/MSI-H solid tumors based on results of the phase I GARNET study which included 327 dMMR/MSI-H solid tumor patients (GC/GEJ, n = 43). In the GC/GEJAC cohort, the ORR of patients treated with dostarlimab was 45.5% (95% CI 24.4 – 67.8%) with a mPFS of 5.5 months (95% CI 2.8 – NE), and mOS of 20.1 months (6.7 – NE) which was very encouraging in this heavily pretreated patient population. Dostarlimab was well tolerated with 11% of patients experiencing grade 3/4 irAE, the most common being hypothyroidism (6.9%) [34].

Patients may also be candidates for pembrolizumab in the second or later line if they have a high tumor mutational burden (TMB-H), defined as ≥ 10 mutations/megabase (mut/mb), which has been associated with better response to ICI and may occur in up to 9% of EAC patients and 29% of GC patients [42]. Though median TMB varies within each solid tumor type, patients with high TMB according to tumor (highest 20% in each cancer type) have been shown to have improved OS after receipt of ICI [43]. In the phase II KEYNOTE-158 trial, 102 patients (13.0%) with TMB-H solid tumors were treated with pembrolizumab with an ORR of 29%. The 87% of patients with TMB < 10 mut/mb treated with pembrolizumab had an ORR of just 6% [44]. After this study, tumor-agnostic approval was granted for pembrolizumab in patients with TMB ≥ 10 mut/mb and may be an option for patients who previously did not progress on ICI in the first-line setting. Though the approval was tumor-agnostic and TMB varies by tumor type, establishing cutoff values for high TMB within each histology may help tailor therapy.

In the third-line or later, trifluridine/tipiracil (TAS-102) is approved based on the phase III TAGS study which enrolled 507 patients with GC or GEJ cancer (GEJ, n = 145) to trifluridine/tipiracil or placebo. With an improved mOS of 5.7 months compared to 3.6 months (HR 0.69 [95% CI0.56 – 0.85]; p = 0.0003) and mPFS of 2 versus 1.8 months (HR 0.57 [95% CI 0.47 – 0.70]; p < 0.0001), this difference was most noticeable in the GC subgroup compared to GEJ patients (mOS HR 0.75 [95% CI 0.50 – 1.11]) [35]. Poor tolerability often limits the use of this drug.

## Targeted Therapy

### HER-2

#### First Line Therapy

HER-2, a member of the EGFR family, is dysregulated in 19 – 32% of GEJ and EAC [45–48]. Given its role in cellular proliferation, migration, and tumorigenesis, as well as its role in breast cancer, targeting HER-2 became an appealing therapeutic option [49]. The initial phase III ToGA trial in 2010 enrolled 584 patients (GC, n = 478; GEJ, n = 106) with HER-2 overexpression by IHC or amplification by FISH to either trastuzumab (HER-2 targeted mAb) with chemotherapy (FP or cisplatin + capecitabine) or chemotherapy alone in the first-line setting. The majority of patients were FISH + /IHC2 + (27%) and FISH + /IHC3 + (44%) and were treated with up to 6 cycles of chemotherapy + placebo or 6 cycles of chemotherapy + trastuzumab followed by maintenance trastuzumab until progression or toxicity. There was a significant improvement in mOS (13.8 vs 11.1 months; HR 0.74 [95% CI 0.60 – 0.91]; p = 0.0046) and mPFS (6.7 vs 5.5 months; HR 0.71 [95% CI 0.59 – 0.85]; p = 0.002) in the trastuzumab arm. Improved OS was especially prominent in the FISH + /IHC2 + and IHC3 + subgroups (mOS 16.0 vs 11.8 months; HR 0.65 [95% CI 0.51 – 0.83]). AEs were similar between cohorts, with rare grade 3/4 cardiac events in both arms (1% with trastuzumab, 3% chemotherapy alone) [49].

Given the benefit of trastuzumab, other HER-2 approaches were then tested. The phase III JACOB trial evaluated dual HER-2 blockade with pertuzumab in addition to chemotherapy compared to trastuzumab plus chemotherapy. No OS difference was detected, so the standard of care was unchanged until the addition of immunotherapy [50]. The role of HER-2 targeting TKIs such as lapatinib with chemotherapy in the front-line setting has been previously explored. The phase III trial TRIO-013/LOGiC evaluated lapatinib in combination with CAPOX in 545 patients with HER-2 positive GEJAC. The mOS of 12.2 months with the addition of lapatinib and 10.5 months in the chemotherapy arm were not significantly different (HR 0.91 [95% CI 0.73–1.12]) [51]. With no differences in survival outcomes compared to chemotherapy alone, lapatinib is not recommended in the first-line setting [45, 51].

The addition of immunotherapy to HER-2 targeting agents was theorized to increase response to combination therapy by improving immune infiltration. Based on this rationale, the phase III KEYNOTE-811 study compared first-line pembro or placebo plus trastuzumab plus chemotherapy (fluoropyrimidine plus platinum) in 698 patients with advanced or metastatic HER-2 positive GC (n = 466) or GEJ cancer (n = 232). In each cohort, 85% of patients had CPS ≥ 1, and the majority were FISH + /ICH2 + (21%) or IHC3 + (78%). The overall analysis showed a mOS (20 vs 16.8 months; HR 0.84 [95% CI 0.70 – 1.01]) and mPFS (10 vs 8.1 months; HR 0.73 [95% CI 0.61 – 0.87]) *that was not significantly improved between cohorts*. However, In the CPS ≥ 1 group, there was improved mOS (20.0 vs 15.7 months; HR 0.81 [95% CI 0.67 – *0.98*]). The ORR was 72.6% compared to 59.8% with the addition of pembrolizumab with a median DOR of 11.2 months (compared to 9 months), and in patients with a treatment response, 31% responded for at least 2 years [52]. Given these findings, the addition of pembrolizumab is approved and recommended for patients who are HER-2 positive and with CPS ≥ 1 [37]. Though patients with EAC were not included in KEYNOTE-811, the findings from GC and GEJ cancer are often extrapolated and applied to patients with advanced or metastatic HER-2 positive EAC.

#### Second or Later Line Therapy—HER-2 Positive

Both TKIs and HER-2 directed antibody therapies other than trastuzumab have been evaluated in the second line setting. Lapatinib was evaluated in the second or later line in the phase III TyTAN study in which 273 patients with advanced GC received either lapatinib plus taxane or taxane monotherapy. Again, the primary endpoint of OS was not met with the addition of lapatinib, though there was a trend towards improved outcomes in patents with HER-2 IHC3 + [53].

The phase II/III GATSBY study evaluated the role of the antibody drug conjugate (ADC) trastuzumab emtansine (T-DM1) in the second or later line from its success in breast cancer [54, 55]. T-DM1 is an antibody drug conjugate that targets the HER-2 receptor by delivering a *tubulin inhibitor* payload. Though T-DM1 was well tolerated, the primary endpoint of OS was not met when compared to taxane monotherapy in this patient population, of whom 77% had received trastuzumab previously. Importantly, the study did not require repeat biopsy on progression and so HER-2 status prior to second-line therapy was unknown [56]. Another study, T-ACT, demonstrated that at time of progression, up to 69% of patients with previously HER-2 positive disease no longer expressed HER-2, demonstrating the importance of confirming HER-2 positivity to guide therapy [45, 57].

The phase II DESTINY-Gastric01 trial done in Asia compared the efficacy of trastuzumab deruxtecan (T-DXd) in the third-line after trastuzumab-containing regimen to chemotherapy and found significantly improved OS with T-DXd [58]. T-DXd differs from T-DM1 in that the payload is a *topoisomerase I inhibitor* which can affect neighboring cells by diffusion causing bystander cell damage. This is especially useful in heterogenous HER-2 positive tumors which may not require a high HER-2 expression for efficacy. Given these findings, a phase III study was designed to enroll HER-2 positive GC or GEJ patients to receive T-DXd in the *second line* after trastuzumab-containing regimen or ram plus paclitaxel [59]. This study is ongoing, the results of which are anticipated in 2025. Another ongoing phase II study, DESTINY-Gastric03, is combining T-DXd with chemotherapy, immunotherapy, or chemoimmunotherapy in both the first and second-line to evaluate safety and response. Importantly, DESTINY-Gastric03 includes both GEJ and EAC patients, the results of which are anticipated in 2026 [60].

### FGFR2b

Fibroblast growth factor (FGF) and its receptor (FGFR) play a role in tumorigenesis by promoting cell growth and angiogenesis via downstream signaling mediated by JAK/STAT, MAPK, and PI3K [61]. The FGFR2b isoform is overexpressed in up to 30% of GC/GEJ and typically portends a poor prognosis [62–64]. Given the on-target effects of pan-FGFR TKIs such as hyperphosphatemia and ocular toxicity, bemarituzumab (bema, FGFR2b mAb) was developed to decrease potential side effects. In a phase I study, patients with GEC were divided into cohorts based on FGFR2b expression. The most benefit from bema was seen in the cohort with IHC 3 + in ≥ 10% of cells [65]. This prompted the phase II FIGHT study which enrolled patients with HER-2 negative GC/GEJ (n = 137; GEJ, n = 18) and FGFR2b overexpression either by ctDNA or IHC 2 + or 3 + in > 10% of cells. Cohorts were randomized to FOLFOX + bema or FOLFOX alone in the first-line. In the overall population, the primary endpoint of significantly improved PFS was not met with the addition of bema, though it was numerically improved (9.5 vs 7.4 months; HR 0.68 [95% CI 0.44 – 10.4]; p – 0.073) [63]. The cohort with FGFR2b overexpression in ≥ 10% of cells (n = 96) showed the most improvement in OS (HR 0.52 [95% CI 0.31 – 0.85]), and in an updated analysis, the mOS trended towards significance with the addition of bema (19.2 vs 13.5 months; HR 0.77 [95% CI 0.52 – 1.14]) in this study with a limited sample size [62]. The addition of bema to FOLFOX was tolerated well, with the exception of increased grade 3/4 cornea disorders with bema (24% vs 0%) [63]. The ongoing phase Ib/III trial, FORTITUDE-102, has recently reported safety data during the phase Ib portion of combination FOLFOX + bema + nivo in 8 GC/GEJ patients with advanced disease in the front-line setting. The best response was PR in 3 patients and SD in 4 patients with no new safety concerns with the combination [66]. The results of the ongoing phase III portion (NCT05111626) enrolling patients with IHC expression of 2 + /3 + in ≥ 10% of tumor cells is expected in 2026 [67].

### FGFR

Fibroblast growth factor/fibroblast growth factor receptor (FGF/FGFR) is associated with cellular proliferation, angiogenesis, and immune suppression within the tumor microenvironment [61]. Alterations in the FGF/FGFR pathway are found frequently in GC and may be caused by point mutations (FGFR4 in 57% of GC), amplifications (2–9%), fusions (20%), and splicing alterations (1.2%) [68]. Targeted agents such as futibatinib/TAS-120, an FGFR1-4 inhibitor, showed promising efficacy in 9 patients with FGFR-altered GC in a phase I trial. In the GC cohort, the ORR was 22.2% (95% CI 2.8 – 60%) with DCR of 55.6% (95% CI 21.2 – 86.3%) [69]. In EC, the combination of futibatinib plus pembro was studied in a phase Ib trial which enrolled patients to futibatinib plus pembro in the second-line or futibatinib, pembro, and chemotherapy (FP or FOLFOX) in the first-line. In ICI-naïve patients treated with futibatinib and pembro, the ORR was 39% with DCR of 75%. Patients who had previously received ICI had an ORR of 6% with DCR of 57% [70]. The response from patients treated in the first-line with futibatinib, pembro, and chemotherapy are ongoing along with the phase II study (NCT05945823) [71].

### Claudin18.2

Claudin18.2 is a tight junction protein found exclusively in gastric and esophageal mucosa where it is found normally between cellular tight junctions and thus is undruggable [72]. During malignant transformation cellular tight junctions are dysregulated and claudin18.2 is found on the exposed tumor cell surface where it can be targeted [73]. Claudin18.2 expression is retained in malignancy and can be overexpressed in a significant amount of GEJ and EAC primary tumors and their metastases, making it an ideal target to act synergistically with chemotherapy [72, 74]. One study found that 18.4% of 485 EAC primary tumors and their metastatic sites examined overexpressed claudin18.2 (defined as expression of any intensity in > 49% of cells). Interestingly, in HER-2 positive tumors, there was significantly decreased overexpression of claudin18.2 (p = 0.036) [72]. Similar to HER-2 positive tumors, claudin18.2 and PD-L1 co-expression is significantly decreased (p < 0.05) with co-expression of PD-L1 (defined as CPS ≥ 1) found in just 19.8% of patients with claudin18.2 expression of 2 + in 40% of tumor cells [75]. In GC and GEJ cancer, the incidence of overexpression (IHC positivity in ≥ 75% of cells) of claudin18.2 has been reported from 24–39% and up to 52% with IHC positivity cutoff of ≥ 40% of cells [73, 76–79]. The MONO trial, a phase IIa study which evaluated the efficacy of zolbetuximab (claudin18.2-targeting mAb) monotherapy in patients with advanced EAC, GEJ, and GC found that 90% of patients who achieved a good response had 2 + / 3 + IHC expression of claudin18.2 in ≥ 70% of cells [80]. Based on this, the phase III SPOTLIGHT and GLOW trials selected patients with advanced HER2- GC/GEJ and claudin18.2 expression of 2 + /3 + by IHC in ≥ 75% of tumor tissue to receive either CAPOX + zolbetuximab (GLOW) or FOLFOX + zolbetuximab (SPOTLIGHT) in the first-line setting [81, 82]. *Notably, in the GLOW trial, 78.1% (n* = *225) of patients with available PD-L1 testing had a CPS* < *5.* The GLOW trial met its primary endpoint of significantly improved mPFS in the zolbetuximab + CAPOX cohort compared to CAPOX (8.21 vs 6.8 months; HR 0.69 [95% CI 0.54 – 0.87]; p = 0.0007) along with significantly improved mOS (14.4 vs 12.2 months; HR 0.77 [95% CI 0.62 – 0.97]; p = 0.0118). The ORR rates were similar between groups (42.5% with zolbetuximab vs 40.3%) and there were more grade 3/4 nausea (8.7% vs 2.4%), vomiting (12.2% vs 3.6%), and anorexia (6.7% vs 1.6%) with the addition of zolbetuximab. Though fewer GEJ patients (n = 35 received zolbetuximab + CAPOX) were enrolled, a subgroup analysis did not show a statistically significant benefit in PFS (HR 1.35 [95% CI 0.73 – 2.5]) or OS (HR 1.01 [95% CI 0.56 – 1.82]) compared to GC [81].

The SPOTLIGHT trial (N = 565; GEJ, n = 136) similarly met its primary endpoint of significantly improved mPFS (10.6 vs 8.7 months; HR 0.75 [95% CI 0.60 – 0.94]; p = 0.0066) with the addition of zolbetuximab to FOLFOX compared to FOLFOX alone. The mOS was also longer with the addition of zolbetuximab (18.2 vs 15.5 months). The GEJ cohort showed no significant difference in PFS outcomes (HR 1.02 [95% CI 0.65 – 1.59]) compared to GC but due to the small sample size of GEJ patients, the trial was not powered for comparison within subgroups. Expected grade 3/4 AEs included more nausea/vomiting (16% vs 6%) in the zolbetuximab + FOLFOX cohort [82]. Zolbetuximab has shown promising efficacy and is undergoing review by the FDA to be approved in the near future [83]. The targeted treatment approaches reviewed here are highlighted in Table 2.

**Table 2:** Clinical trials highlighting targeted therapy in the first and second-line setting

NCT/Reference	Trial	Study Agent	Study Population	Primary Outcome	mPFS (months)	HR (95% CI) p-value	mOS (months)	HR (95% CI) p-value	ORR (%)
*HER-2 Targeted Therapy First Line*									
NCT01041404 [49]	ToGA	Trastuzumab + chemo (FP or Cisplatin + capecitabine) vs chemo	1st line GC, GEJAC with HER2 -overexpression	OS	mPFS: 6.7 vs 5.5	0.71 (0.59, 0.85) p = 0.0002	13.8 vs 11.1	0.74 (0.60, 0.91) p = 0.0046	47% vs 35%
NCT01774786 [50]	JACOB	Pertuzumab + Trastuzumab + chemo (Cisplatin, capecitabine, or 5FU) vs Trastuzumab + chemo	1st line HER2-positive GC, GEJAC	OS	mPFS: 8.5 vs 7.0	0.73 (0.62, 0.86) p = 0.0001	17.5 vs 14.2	0.84 (0.71, 1.00) p = 0.057	56.7% vs 48.3%
NCT00680901 [51]	TRIO-013/LOGiC	Lapatinib + CapeOx vs CapeOx + placebo	1st line HER-2 positive GC, EAC, or GEJAC	OS	mPFS: 6.0 vs 5.4	0.82 (0.68, 1.00) p = 0.0381	12.2 vs 10.5	0.91 (0.73, 1.12)	53% vs 39%
NCT03615326 [52]	KEYNOTE-811	pembro + Trastuzumab + chemo (5FU + platinum) vs Trastuzumab + chemo	1st line HER2- positive GC or GEJAC	PFS and OS	mPFS: 10.0 vs 8.1 CPS ≥ 1 group: 10.9 vs 7.3	0.72 (0.60, 0.87) p = 0.0002 CPS ≥ 1 group: 0.71 (0.59, 0.86)	20.0 vs 16.8 CPS ≥ 1 group: 20.0 vs 15.7	0.84 (0.70, 1.01) CPS ≥ 1 group: 0.81 (0.67, 0.98)	72.6% vs 59.8%
*HER-2 Targeted Therapy Second or Later Line*									
NCT00486954 [53]	TyTAN	Lapatinib + paclitaxel vs paclitaxel	2nd line, HER2- positive GC	OS	mPFS: 5.4 vs 4.4	0.85 (0.63, 1.13) p = 0.2441	11.0 vs 8.9	0.84 (0.64, 1.11) p = 0.1044	27% vs 9%
NCT01641939 [56]	GATSBY	Trastuzumab emtansine vs docetaxel or paclitaxel	2nd line, HER2-positive GC	OS	mPFS: 2.7 vs 2.9	1.13 (0.89, 1.43) p = 0.31	7.9 vs 8.6	1.15 (0.87, 1.51) p = 0.86	20.6% vs 19.6%
NCT03329690 [84]	DESTINY-Gastric01	Trastuzumab deruxtecan vs chemotherapy	3rd line, HER2-positive GC, GEJAC	ORR	mPFS: 5.6 vs 3.5	0.47 (0.31, 0.71)	12.5 vs 8.4	0.59 (0.39, 0.88) p = 0.01	51% vs 14% p < 0.001
*FGFR2b*									
NCT03694522 [63]	FIGHT	Bemarituzumab + mFOLFOX6 vs placebo + mFOLFOX6	1st line FGFR2b-positive, HER-2 negative GC, GEJAC	PFS	mPFS: 9.5 vs 7.4	0.72 (0.49, 1.08)	19.2 vs 13.5	0.77 (0.52, 1.14)	56.5% vs 36.5%
*Claudin18.2*									
NCT01197885 [80]	MONO	zolbetuximab	2nd line or later GC, GEJAC, EAC with moderate-to-strong CLDN18.2 expression in 50% of tumor cells	ORR					Allcomers: 9% CLDN18.2 expression ≥ 70%: 14%
NCT03504397 [82]	SPOTLIGHT	Zolbetuximab + mFOLFOX6 vs placebo + mFOLFOX6	1st line HER2-negative, CLDN18.2-positive GC, GEJAC	PFS	mPFS: 10.6 vs 8.7	0.75 (0.60, 0.94) p = 0.0066	18.2 vs 15.5	0.75 (0.60, 0.94) p = 0.0053	48% vs 48%
NCT03653507 [81]	GLOW	zolbetuximab + CAPOX vs placebo + CAPOX	1st line HER2-negative, CLDN18.2-positive GC, GEJAC	PFS	mPFS: 8.21 vs 6.80	0.69 (0.54, 0.87) p = 0.0007	14.4 vs 12.2	0.77 (0.62, 0.97) p = 0.0118	42.5% vs 40.3%

Trials highlighting targeted therapy and combination therapy. Subgroup analysis shown in certain trials to highlight mutational profile. Abbreviations: *HR* hazard ratio, *mOS* median overall survival, *mPFS* median progression free survival, *ORR* objective response rate

## Emerging and Novel Treatment Approaches

### TIGIT/PD-1 Blockade

T-cell Ig and ITIM domain (TIGIT) is present on activated T-cells and natural killer (NK) cells and when activated, decreases T-cell and NK cell function to dampen the immune response [85]. TIGIT and PD-L1 inhibitors together act synergistically to remove immune system checkpoints, allowing for recognition of neoplastic cells. In a review of 30 patients with EC, TIGIT and PD-1 expression were increased in tumor infiltrating lymphocytes (TILs), preventing robust immune response within the tumor micro-environment [86]. In a phase Ib study of atezolizumab (anti-PD-L1 mAb) with tiragolumab (anti-TIGIT mAb) in metastatic EC (N = 21; EAC, n = 7; ESCC, n = 13), no new safety signals were appreciated (grade 1–3 irAE in 57.1%), and the ORR was 27.8% in this heavily pretreated population [87]. Subsequently, the phase II EDGE-Gastric trial (ongoing) reported the results of arm A1 which enrolled patients with metastatic GC/GEJ/EAC to domvanalimab (dom, anti-TIGIT mAb) plus zimberelimab (zim, anti-PD-1 mAb) plus FOLFOX in the first-line. Of the 41 patients enrolled at the time of data cutoff (at least 2 imaging assessments), 76% continued on therapy with an ORR of 59% (95% CI 42%—74%). Patients with higher PD-L1 expression by tumor area positivity (TAP, defined as the proportion of PD-L1 positive tumor cells and immune cells in the tumor area) ≥ 5 derived the most ORR (80% vs 46%) and 6-mo PFS rate benefit (93% vs 66%) compared to patients with TAP < 5% [88]. The confirmatory phase III STAR-221 trial (NCT05568095) is enrolling patients with metastatic GC/GEJ/EAC 1:1 to dom plus zim plus FOLFOX or nivo plus FOLFOX with results expected in 2027 [89]. While TIGIT/PD-1 blockade may soon add to the armamentarium of first-line treatments for patients, future results will reveal if the benefit is driven by those with higher PD-L1 expression as determined by TAP.

### Bispecific and Novel Monoclonal Antibodies

#### Immunomodulatory BsAb

Bispecific antibodies (BsAb), successful in hematologic malignancies, are in development for solid tumors. They harness the immune system by pairing by proximity an immune effector mechanism with either a targetable cancer antigen or another immune cell [90].

#### TIGIT

TIGIT/PD-1 BsAb, such as rilvegostomig, are active in solid tumors after tumor progression on single-agent ICI. This is under study in solid tumors, including GEJ and EAC, in a multi-arm phase II trial (NCT05702229) [91, 92]. Others include CTLA-4/PD-1 BsAb and PD-1/TIM-3 BsAb in combination with chemotherapy [92]. The immunosuppressive tumor microenvironment of immune “cold” tumors may limit the efficacy of these novel treatment approaches although it remains to be seen if BsAb can help improve efficacy [93].

#### Claudin18.2

Bispecific and trispecific antibodies that link claudin18.2 to an immune effector cells and T-cells are also under study [94]. In a phase Ia/Ib trial, Q-1802, a BsAb targeting claudin18.2 and PD-1, is under study in patients with metastatic solid tumors who progressed on usual therapy. The phase Ia results from the initial 9 patients (majority with GI malignancies) who received Q-1802 showed that it was safe and tolerable, with the most common AEs being GI (nausea, vomiting, diarrhea) [95].

Osemitamab (TST001) targets claudin18.2 by activating NK cell-mediated cytotoxicity. It is a novel mAb which works to activate antibody-dependent cellular cytotoxicity via Fc binding to NK cells. Its increased NK-binding affinity allows it to be more effective in patients with low or medium claudin18.2 expression [96]. The ongoing phase I/IIa TranStar101 study in patients with advanced GC/GEJAC included osemitamab monotherapy in the dose-escalation portion, osemitamab plus nivo in the second or later line, or osemitamab plus nivo plus FOLFOX in the first line. Safety is acceptable and accrual to all cohorts is ongoing [96]. TranStar102 previously reported safety and efficacy data in 64 patients with claudin18.2 IHC expression of 1 + in ≥ 10% of cells, confirming the hope that patients with low or medium claudin18.2 expression may benefit from therapy. In the 40 patients who received osemitamab plus CAPOX in the first-line, preliminary results showed 27 achieved a PR with ORR, PFS, and DOR assessment ongoing [97].

#### HER-2

Zanidatamab (zani), a BsAb targeting two domains of HER-2, is also under study in two trials. This HER-2 targeting BsAb works by increasing extracellular binding, internalization, and complement-mediated destruction, thus allowing for greater tumor-mediated toxicity [98]. One study is a phase II trial (NCT03929666) of zani in combination with standard first-line chemotherapy [99]. The other is a phase I trial in combination with a PD-1 inhibitor (NCT04276493) [100]. Results are awaited.

Other HER-2 mAbs currently under study in first-line in combination with either PD-1 inhibition or BsAb include margetuximab and tebotelimab. Margetuximab is a HER-2 targeted mAb, and the BsAb tebotelimab targets PD-1 and LAG-3. The phase II/III MAHOGANY trial (NCT04082364) treats HER-2 positive GC or GEJAC patients with margetuximab plus retifamlimab (anti-PD-1 mAb) with/without chemotherapy and margetuximab plus tebotelimab with chemotherapy. By inhibiting both PD-1 and LAG-3 while targeting HER-2, the goal is to re-activate the immune system within HER-2 positive tumor cells [101].

#### Antibody–Drug Conjugates

Antibody–drug conjugates (ADCs) are a monoclonal antibody that target delivery of a cytotoxic payload (smart bomb). An example of this is T-DXd, which targets HER-2, which was discussed earlier. The goal is to minimize off-target effects of the chemotherapy while also modulating the tumor microenvironment to create a multipronged anti-tumor effect [102]. Several ADCs targeting proteins which are often overexpressed in GC/GEJ/EAC are currently underway and highlighted here.

#### Claudin18.2

CMG901, a claudin18.2-ADC with the antimitotic payload, monomethyl auristatin E, was studied in a phase I trial. It led to an ORR of 75% and DCR of 100% in 13 heavily pretreated claudin18.2-positive GC/GEJAC patients. Maturation of the PFS and OS data and the phase Ib portion of the study are ongoing [103]. In China and Australia, the CLINCH study, evaluating claudin18.2-ADC ATG-022 monotherapy in patients with pretreated advanced GC, was just expanded to the phase II portion [104]. The FDA recently granted an orphan drug approval to ATG-022 in patients with claudin18.2 positive GC and pancreas adenocarcinoma [105]. Several other clinical trials evaluating the use of claudin18.2-ADCs in solid tumors expressing claudin18.2 are ongoing (RC118, NCT05205850; SKB315, NCT05367635) [106, 107].

#### TROP2

TROP2, found normally on epithelial cells and not highly expressed in/on gastrointestinal cells, is strongly overexpressed in up to 13% of GC and 24% of EAC [108, 109]. Sacituzumab govitecan is an ADC that delivers an irinotecan-derived (topoisomerase I inhibitor) payload to TROP2-positive cells. The ongoing phase Ib/II SAGA trial is evaluating the efficacy of this TROP2-ADC in patients with advanced EAC/GEJ/GC. It excludes patients with prior topoisomerase inhibitor exposure. Outcomes will be stratified by TROP2 expression [109].

Sacituzumab tirumotecan (SKB264) is a TROP2-ADC which has a topoisomerase I inhibitor attached by a novel linker that release the cytotoxic payload based on extracellular pH changes, which are hypothesized to increase payload efficacy. Preliminary results from an ongoing phase II study enrolling heavily pretreated GC/GEJAC patients (N = 48) treated with SKB246 showed an ORR of 22.0%, DCR of 80.5%, and DOR of 7.5 months. There were no new safety concerns, including no pneumonitis or neuropathy, and the most common grade 3/4 AEs were hematologic [110]. A phase III trial is planned and correlation with TROP2-expression may further elucidate which patients may respond best to this novel therapy.

#### HER-2

The development of T-DXd, an FDA approved HER-2 ADC, opened the field for study of ADCs with increased payload activity. SHR-A1811, links a novel topoisomerase I inhibitor to trastuzumab. The payload, SHR9265, has shown increased membrane permeability for delivery optimization to HER-2 positive cells. It also delivers bystander drug to HER-2 low or negative cells—which is ideal given the intra-tumoral heterogeneity of HER-2 in gastroesophageal malignancies [111]. The phase I study including 98 patients with HER-2 expressing or positive (defined as IHC3 + or 2 + with ISH +) colorectal cancer (n = 43; HER-2 positive, n = 32) and GC/GEJ (n = 55; HER-2 positive, n = 32) who received SHR-A1811 reported an ORR of 43.8% (95% CI 26.4 – 62.3) in the HER-2 positive GC/GEJ cohort. The 6-month PFS was 73.9% (95% CI 52.1 – 86.9). Grade 3/4 AEs that occurred in ≥ 10% of patients were hematologic with no reports of interstitial lung disease or pneumonitis [112]. A phase III trial (NCT06123494) comparing SHR-A1811 versus investigator’s choice is ongoing in the second or later line for HER-2 positive GC/GEJAC who have progressed after prior HER-2 targeted regimens [113].

Disitamab vedotin (RC48), is another anti-HER-2 antibody conjugated with a microtubule inhibitor. A single-arm phase II study evaluated disitamab vedotin as second-line treatment for 125 eligible patients with HER-2 overexpressing GC or GEJ cancer (IHC 2 + /3 + and IHC 2 + FISH-). The ORR was 24.8% (95% CI 17.5–33.3), with a median PFS of 4.1 months (95% CI 3.7–4.9) and OS of 7.9 months (95% CI 6.7–9.9). Grade 3/4 AEs were most commonly hematologic, and interstitial lung disease and pneumonitis were not observed [114].

### CAR-T and CAR-M

Chimeric Antigen Receptor T-cell therapy (CAR-T) is emerging as a treatment modality for solid tumors, and many/most of the known drug targets reviewed above are being approached in this way. CAR-T therapy manipulates the T-cell receptor (TCR) in patient-derived T-cells in a bespoke way, such that they recognize a selected cancer associated antigen. When the engineered TCR is expressed together with co-activating signals, direct activation of T-cells may occur in the absence of antigen presenting cells.

One example is novel claudin18.2-directed CAR-T, CT041, which was evaluated in GC and pancreas adenocarcinoma in a phase Ib study. Interestingly, GC patients had the best responses even after > 3 prior lines of therapy. Of the 3 GC patients included, the ORR was 100% (1 CR, 2 PR) [115]. A phase Ib/II study is enrolling previously treated GC/GEJAC patients to receive CT041 [116]. Preliminary results from the phase Ib portion including 14 patients showed PR in 57.1% and SD in 14.3% of patients with a mPFS of 5.8 months (95% CI 1.9 – 7.4) and mOS of 10.8 months (95% CI 5.1 – NE). The majority of grade 3/4 AEs were hematologic and only one patient experienced grade 4 cytokine release syndrome but completely recovered. The phase II portion remains ongoing [116]. LB1908, another claudin18.2-directed CAR-T, is under study in an ongoing phase I trial (NCT05539430) including patients with EAC/GEJAC/GC and pancreas adenocarcinoma with claudin18.2 IHC ≥ 1 + in ≥ 50% of cells [117].

Activated macrophages upregulate presentation of tumor-associated antigens to activate cytotoxic T-cells. Chimeric Antigen Receptor-Macrophages (CAR-M) are not only able to recognize tumor antigens and phagocytose neoplastic cells, but also cause cytokine release to induce an inflammatory milieu which propagates an immune response in the tumor microenvironment [118]. A phase I trial is investigating CT-0508, an anti-HER-2 CAR-M in HER-2 overexpressing (IHC 3 + or 2 + /FISH +) solid tumors either alone or with ICI (NCT04660929) [119].

As more potentially targetable cancer antigens are discovered in GEJ/EAC and providers become comfortable managing the potential side effects, we anticipate further CAR-T and CAR-M directed trials towards tumor-associated antigens.

**Fig. 1:**
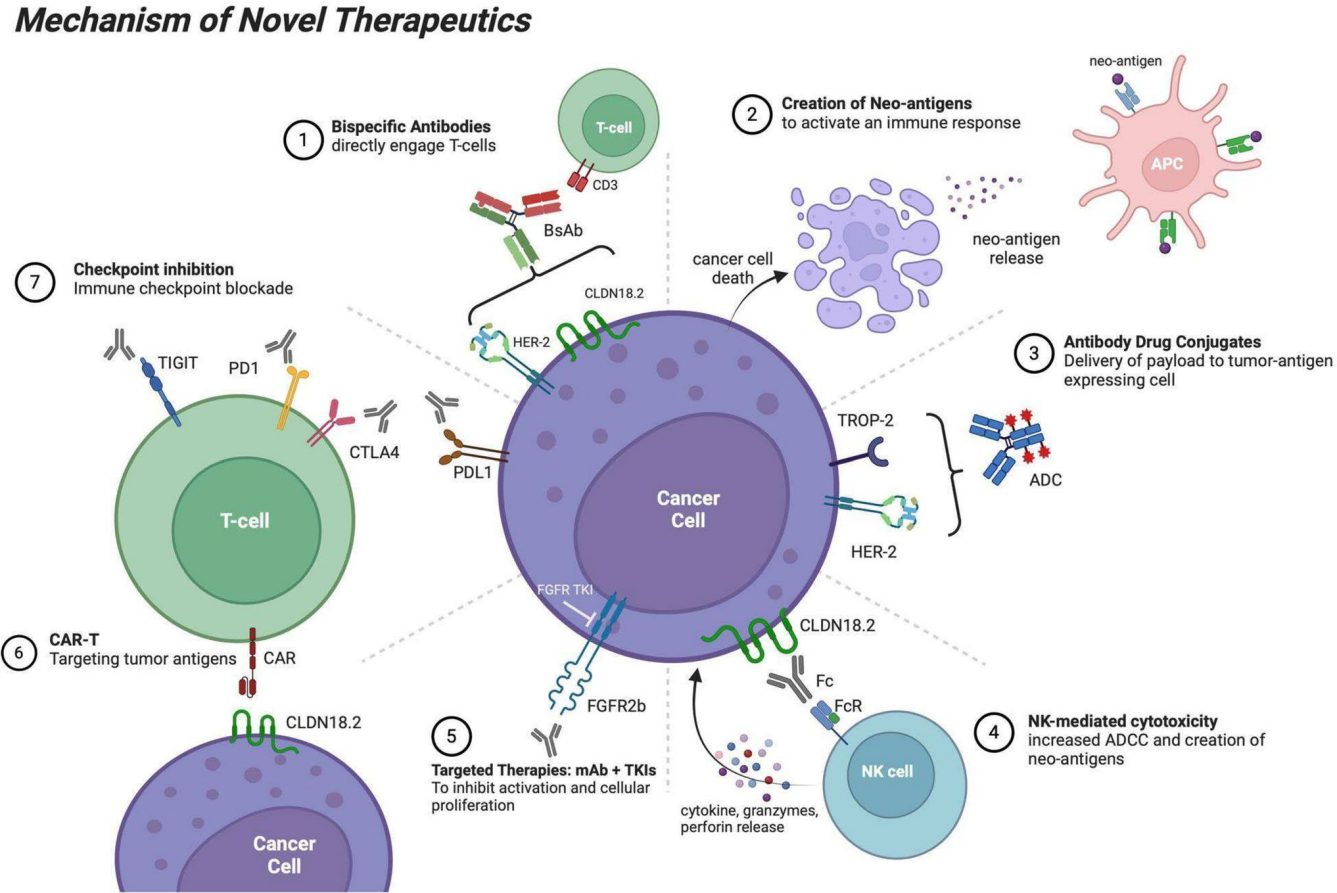
Mechanism of Novel Therapeutics displays the different mechanisms of action of drug targets in development for gastroesophageal adenocarcinoma. Bispecific antibodies (1) join specific cancer antigens, such as CLDN18.2 and HER-2, to cytotoxic T-cells for destruction. Destruction by T-cells and NK cells via ADCC (4) leads to the release of cancer antigens which further stimulate the immune response within the tumor microenvironment (2). Antibody drug conjugates (3) are tumor-targeted antibodies attached to a cytotoxic payload which poisons both the antigen-expressing cells and neighboring cancer cells. Targeted monoclonal antibodies and TKIs (5) inhibit tumorigenesis by blocking proliferative cellular pathways. CAR-T (6) allows cytotoxic T-cells to directly recognize cancer antigens via the TCR, causing activation and proliferation of cytotoxic CAR-Ts for tumor destruction. Lastly, immune checkpoint inhibition (7) via CTLA-4, PD1/PD-L1, and TIGIT allows for enhanced immune recognition of tumor cells. Abbreviations: ADC, antibody drug conjugate; ADCC, antibody-dependent cellular cytotoxicity; CAR-T, chimeric antigen receptor T-cell; CLDN18.2, claudin18.2; mAb, monoclonal antibody; TKI, tyrosine kinase inhibitor. Created with BioRender.com

Select novel therapeutic mechanisms of action are featured in Fig. 1.

## Conclusion

EAC and GEJAC are increasingly prevalent and continue to be associated with a poor prognosis. Fortunately, advances in predictive markers have led to an explosion of approaches to target these pathways and mechanisms. This translates into survival benefit in the recurrent/metastatic setting. Application of these approaches which target HER-2, FGFR, claudin18.2, and PD-1 requires molecular profiling of tumors by both next generation sequencing and immunohistochemistry. Within this rapidly changing treatment environment it is critical to get upfront molecular testing to determine selection, combination, and sequencing of these targeted approaches. Due to the heterogenous nature of these tumors, reassessment with biopsy is often needed at progression to re-evaluate mutation profile and direct further therapy. Given the multitude of heterogenous treatment approaches currently under evaluation, early clinical trial enrollment in a setting of multidisciplinary evaluation is key to increasing survival and wellbeing in patients with gastroesophageal cancer.

## Key References

- Janjigian YY, Shitara K, Moehler M, Garrido M, Salman P, Shen L, et al. First-line nivolumab plus chemotherapy versus chemotherapy alone for advanced gastric, gastro-oesophageal junction, and oesophageal adenocarcinoma (CheckMate 649): a randomised, open-label, phase 3 trial. Lancet. 2021;398(10294):27–40. 10.1016/S0140-6736(21)00797-2.
  - This study revolutionalized first-line treatment with the addition of immunotherapy
- Sun JM, Shen L, Shah MA, Enzinger P, Adenis A, Doi T, et al. Pembrolizumab plus chemotherapy versus chemotherapy alone for first-line treatment of advanced oesophageal cancer (KEYNOTE-590): a randomised, placebo-controlled, phase 3 study. Lancet. 2021;398(10302):759–71. 10.1016/s0140-6736(21)01234-4.
  - Keynote-590 continued to show benefit with the addition of immunotherapy in the first-line setting for qualifying patients.
- Rha SY, Oh DY, Yañez P, Bai Y, Ryu MH, Lee J, et al. Pembrolizumab plus chemotherapy versus placebo plus chemotherapy for HER2-negative advanced gastric cancer (KEYNOTE-859): a multicentre, randomised, double-blind, phase 3 trial. Lancet Oncol. 2023;24(11):1181–95. 10.1016/s1470-2045(23)00515-6.
  - Addition of immunotherapy in the first-line setting improves survival outcomes for qualifying patients.
- Janjigian YY, Kawazoe A, Bai Y, Xu J, Lonardi S, Metges JP, et al. Pembrolizumab plus trastuzumab and chemotherapy for HER2-positive gastric or gastro-oesophageal junction adenocarcinoma: interim analyses from the phase 3 KEYNOTE-811 randomised placebo-controlled trial. Lancet. 2023;402(10418):2197–208. 10.1016/S0140-6736(23)02033-0.
  - Combination immunotherapy with targeted HER-2 therapy showed improved outcomes in HER-2 positive patients, changing the first-line treatment paradigm.
- Shitara K, Bang Y-J, Iwasa S, Sugimoto N, Ryu M-H, Sakai D, et al. Trastuzumab Deruxtecan in Previously Treated HER2-Positive Gastric Cancer. New England Journal of Medicine. 2020;382(25):2419–30. 10.1056/NEJMoa2004413.
  - Patient with HER-2 positive disease benefit from T-DXd even after HER-2 targeted agents.
- Shah MA, Shitara K, Ajani JA, Bang YJ, Enzinger P, Ilson D, et al. Zolbetuximab plus CAPOX in CLDN18.2-positive gastric or gastroesophageal junction adenocarcinoma: the randomized, phase 3 GLOW trial. Nat Med. 2023;29(8):2133–41. 10.1038/s41591-023-02465-7.
  - The addition of targeted CLDN18.2 therapy to standard of care first-line chemotherapy improved outcomes for select patients.
- Shitara K, Lordick F, Bang YJ, Enzinger P, Ilson D, Shah MA, et al. Zolbetuximab plus mFOLFOX6 in patients with CLDN18.2-positive, HER2-negative, untreated, locally advanced unresectable or metastatic gastric or gastro-oesophageal junction adenocarcinoma (SPOTLIGHT): a multicentre, randomised, double-blind, phase 3 trial. Lancet. 2023;401(10389):1655–68. 10.1016/s0140-6736(23)00620-7.
  - The addition of targeted CLDN18.2 therapy to standard of care first-line chemotherapy improved outcomes for select patients.
- Janjigian YY: EDGE-Gastric Arm A1: Phase 2 study of domvanalimab, zimberelimab, and FOLFOX in first-line (1L) advanced gastroesophageal cancer. https://meetings.asco.org/abstracts-presentations/228836 (2024). Accessed May 1 2024.
  - Combination immune checkpoint inhibition with chemotherapy shows good safety profile and potentially improved outcomes in the first-line setting.

## Data Availability

No datasets were generated or analysed during the current study.
